# Synthesis and Biological Evaluation of Some [1,2,4]Triazolo[4,3-*a*]quinoxaline Derivatives as Novel Anticonvulsant Agents

**DOI:** 10.1155/2013/587054

**Published:** 2013-09-12

**Authors:** Mohamed Alswah, Adel Ghiaty, Ahmed El-Morsy, Kamal El-Gamal

**Affiliations:** Faculty of Pharmacy, Organic Chemistry Department, Al-Azhar University, Cairo 11884, Egypt

## Abstract

2-([1,2,4]Triazolo[4,3-*a*]quinoxalin-4-ylthio)acetic acid hydrazide (**10**) was used as a precursor for the syntheses of novel quinoxaline derivatives with potential anticonvulsant properties. The newly synthesized compounds have been characterized by IR, ^1^H NMR, and mass spectral data followed by elemental analysis. The anticonvulsant evaluation was carried out for eleven of the synthesized compounds using metrazol induced convulsions model and phenobarbitone sodium as a standard. Among this set of tested compounds, two of them (**14**, and **15b**) showed the best anticonvulsant activities.

## 1. Introduction

Syntheses of quinoxalines have attracted a great deal of attention in view of their potent biological and pharmacological activities including anticonvulsant [1–4], antibacterial [5], antifungal [6], antiviral [7], antitubercular [8], antileishmanial [9], antiamoebic [10], analgesic [11], antihistaminic [12], antineoplastic [13], hypoglycemic [14], MAO-A inhibitor [15], antiarrhythmic [16], antiatherosclerotic [17], antiobese [18], and other diverse pharmacological activities.

Earlier studies revealed that most of compounds derived from 1,2,4-triazoles have been found to be significant anticonvulsant [19] and tranquillizing agents [20]. 

**Figure 1:**
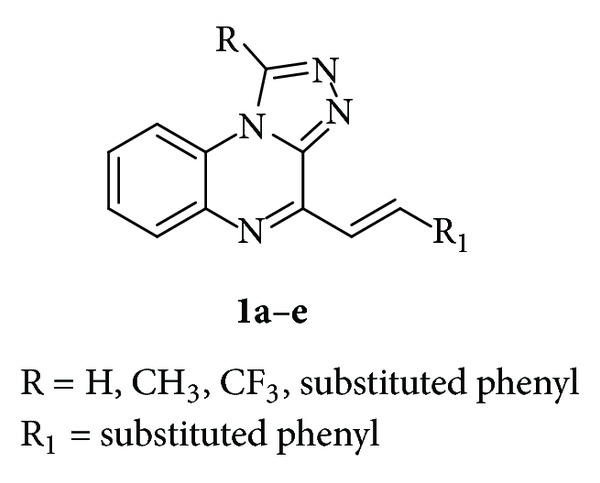


Furthermore, compounds **1a**–**e** in Figure 1 which contain [1,2,4]triazolo[4,3-*a*]quinoxaline moiety showed promising anticonvulsant activity [21].

We describe the synthesis and biological evaluation of novel [1,2,4]triazolo[4,3-*a*]quinoxaline derivatives expected to have anticonvulsant activity starting from 1,2-diaminobenzene and oxalic acid via quinoxaline ring build-up.

## 2. Results and Discussion

### 2.1. Chemistry

**Scheme 1:**
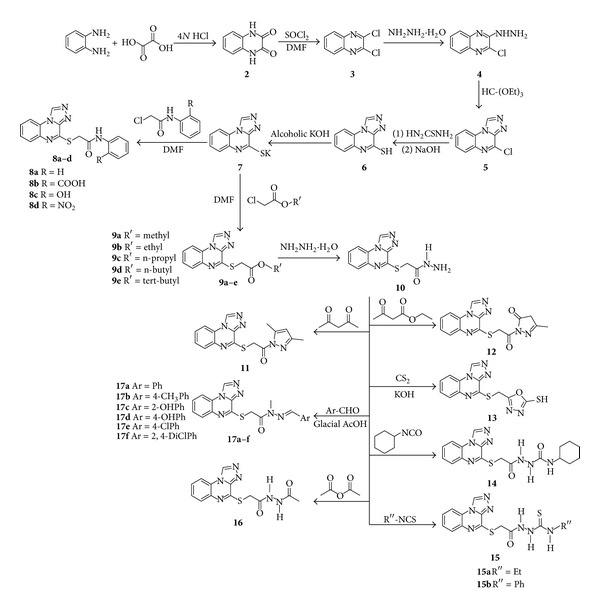


The [1,2,4]triazolo[4,3-*a*]quinoxaline derivatives were prepared using established methodology as shown in Scheme 1. 2,3-Dichloroquinoxaline (**3**) was prepared by chlorination of 2,3-dihydroxyquinoxaline (**2**), which in turn was prepared by the condensation of the commercially available 1,2-diaminobenzene with oxalic acid in aqueous hydrochloric acid. Treatment of **3** with hydrazine hydrate yielded the corresponding 3-hydrazino compound, **4**, which was subjected to ring closure to **5** by treatment with triethyl orthoformate. Reaction of **5** with thiourea in absolute ethanol afforded the isothiouronium intermediate which upon basic hydrolysis yielded [1,2,4]triazolo[4,3-*a*]quinoxaline-4-thiol (**6**). The potassium salt **7** was obtained after treatment of **6** with alcoholic KOH in a quantitative yield.

Reaction of the potassium salt **7** with substituted aniline in DMF gave the corresponding anilide **8a**–**d**, and its reaction with alkyl chloroacetate in the same solvent gave the corresponding ester **9a**–**e**. The hydrazide derivative **10** was synthesized from the reaction of n-butyl ester with hydrazine hydrate. The hydrazide **10** was used as the key intermediate for the further syntheses. Thus, that hydrazide was allowed to react with ethyl acetoacetate and acetylacetone to give the corresponding methylpyrazolone derivative **11** and dimethylpyrazole derivative **12**, respectively. When compound **10** was treated with carbon disulphide and potassium hydroxide, oxadiazole derivative **13** was obtained in an excellent yield. Allowing the same intermediate **10** to react with isocyanate or with isothiocyanates resulted in the formation of semicarbazide **14** and thiosemicarbazides **15**, respectively. Acetylation of compound **10** with acetic anhydride and condensation with variety of aromatic aldehydes gave the corresponding *N-*acetyl derivative **16** and *N-*arylidene derivatives **17a**–**f**, respectively. 

The structural assignments to the new compounds were based on their elemental analysis and spectral (IR, ^1^H NMR, and mass) data.

## 3. Biological Activity

### 3.1. Anticonvulsant Activity

Eleven compounds of the newly synthesized derivatives were selected to be screened for their anticonvulsant activity on different groups of mice. Swiss albino adult male mice, weighing 20–25 g, were used as experimental animals. They were obtained from an animal facility (Animal House, Department of Pharmacology and Toxicology, Faculty of Pharmacy, Al-Azhar University). Mice were housed in stainless steel wire-floored cages without any stressful stimuli. Animals were kept under well-ventilated conditions at room temperature (25–30°C). They were fed on an adequate standard laboratory chow (El-Nasr Co., Abou-Zabal, Egypt) and allowed to acclimatize with free access to food and water for 24-hour period before testing except during the short time they were removed from the cages for testing. Albino mice were randomly arranged in groups, each of six animals. Phenobarbitone sodium was used as a reference drug for comparison. Pentylenetetrazole (PTZ, Sigma Chemicals, USA) was used as convulsant.

The tested compounds were dissolved in DMSO and orally administered in a dose regimen ranging from 200 to 800 mg/kg animal weight, using the dosing volume of 0.2 mL per 20 g. Pentylenetetrazole was dissolved in normal saline in 2% concentration and was given intraperitoneally in a dose of 60 mg/kg body weight (dose that could induce convulsions in at least 80% of the animals without death during the following 24 hours). Phenobarbitone sodium (Alex Co., Egypt) was dissolved in normal saline in 2% concentration and it was intraperitoneally given in doses of 6.25, 12.5, and 25 mg/kg using the same dosing volume. All drugs were freshly prepared to the desired concentration just before use.

Groups of six mice were administered the graded doses of the test compounds and phenobarbitone sodium orally. Control animals received an equal volume of saline (10 mL/kg). After one hour, the animals were subcutaneously injected with the convulsive dose of pentylenetetrazole (60 mg/kg). The criterion of anticonvulsant activity is complete protection against convulsions of any kind. Observations were made at least 60 minutes after the administration of pentylenetetrazole. Doses that gave full protection against the induced convulsions and those which exhibited 50% protection in addition to the relative potencies of the test compounds to phenobarbitone sodium were recorded in Table 1. Observations were made at least 60 min after the administration of metrazol.

## 4. Experimental

### 4.1. General

Melting points were determined by open capillary and are uncorrected. The IR spectra (in KBr discs) were recorded on potassium bromide discs on a Pye Unicam SP 3300 spectrophotometer. ^1^H NMR spectra were recorded either on Gemini 300 MHz (Varian USA) or on Jeol ECA 500 MHz NMR spectrometer (500 MHz) using TMS as an internal standard. All chemical shifts are reported in ppm downfield from TMS. Mass spectra were recorded on a Shimadzu GCMS-QP 1000 EX mass spectrometer operating at 70 eV. Elemental analysis was carried out at the Regional Center for Mycology and Biotechnology, Al-Azhar University, Cairo, Egypt. Progress of the reactions was monitored by TLC sheets precoated with UV fluorescent silica gel Merck 60 F254 plates and was visualized using UV lamp and n-hexane : ethyl acetate 9 : 1 as mobile phase. Starting materials were purchased from Aldrich Chemical Company and used without further purification. Compounds **2**–**6** were prepared following the procedures reported in the literature [22–24]. The used intermediates (chloroacetanilides and alkyl chloroacetates) were prepared as reported [25, 26].

#### 4.1.1. Preparation of Potassium Salt of [1,2,4]Triazolo[4,3-a]quinoxaline-4-thiol **(7)**

Compound **6** (1.5 g, 0.01 mol) was treated with alcoholic solution of potassium hydroxide (0.4 g, 0.01 mol); the reaction mixture was stirred for 1 h; the solid obtained was filtered, washed with absolute ethanol, and air-dried to give the potassium salt in quantitative yield. m.p. > 360°C. IR (KBr) cm^−1^: 3391, 3127, 2833, 1629, 1479, 1412, 1309, 1209, 1065, 972. MS (*m/z*, %): 240 (M^+^, 0.9), 233 (4), 202 (6), 160 (7), 143 (42), 118 (34), 102 (11), 91 (22), 77 (24), 63 (100), 51 (27). ^1^H NMR (DMSO-*d*_6_, 300 MHz) **δ**: 7.13 (t, 1H, *J* = 7.50 Hz), 7.27 (t, 1H, *J* = 7.50 Hz), 7.56 (d, 1H, *J* = 7.20 Hz), 8.07 (d, 1H, *J* = 7.20 Hz), 9.77 (s, 1H, triazolo proton). Anal. Calcd. for C_10_H_8_KN_4_S: C, 44.98; H, 2.10; N, 23.31. Found: C, 45.07; H, 2.09; N, 23.38.

#### 4.1.2. Preparation of 2-([1,2,4]Triazolo[4,3-a]quinoxalin-4-ylthio)-N-(substituted phenyl)acetamide **(8a–d)**

General procedure: a mixture of compound **7** (1.0 g, 0.01 mol) and the appropriate chloroacetanilide (0.01 mol) in DMF (20 mL) was heated over a water bath for 3 h. The reaction mixture was then cooled, poured into ice-cooled water (200 mL), and stirred well for 30 min. The solid thus separated was filtered, washed with water, dried, and crystallized from methanol/toluene mixture (1 : 1) to obtain compounds **8a–d**.

*Compound* ***8a***. Yield 0.91 g (68%), m.p. 270–272°C. IR (KBr) cm^−1^: 3216, 3071, 1666, 1599, 1537, 1463, 1318, 1242, 1066, 955. MS (*m/z*, %): 335 (M^+^, 0.1), 243 (1), 215 (2), 203 (11), 143 (14), 132 (25), 119 (22), 104 (14), 91 (100), 77 (44), 64 (82), 50 (26). ^1^H NMR (DMSO-*d*_6_, 500 MHz) **δ**: 4.34 (s, 2H), 7.02 (t, 1H, *J* = 7.56 Hz), 7.26 (t, 2H, *J* = 7.56 Hz), 7.58 (m, 4H), 7.85 (d, 1H, *J* = 8.40 Hz), 8.32 (d, 1H, *J* = 7.65 Hz), 10.09 (s, 1H, triazolo proton). Anal. Calcd. for C_17_H_13_N_5_OS: C, 60.88; H, 3.91; N, 20.88. Found: C, 61.08; H, 3.90; N, 20.94%.

*Compound* ***8b***. Yield 0.93 g (59%), m.p. 238–240°C. IR (KBr) cm^−1^: 3522, 3205, 3122, 1681, 1591, 1459, 1364, 1247, 1057, 957. ^1^H NMR (DMSO-*d*_6_, 500 MHz) **δ**: 4.32 (s, 2H), 7.10 (t, 1H, *J* = 7.65 Hz), 7.43 (m, 4H), 7.89 (d, 2H, *J* = 7.65 Hz), 8.17 (t, 1H, *J* = 7.65 Hz), 10.09 (s, 1H, triazolo proton). Anal. Calcd. for C_18_H_13_N_5_O_3_S: C, 56.98; H, 3.45; N, 18.46. Found: C, 57.19; H, 3.44; N, 18.51%. 

*Compound* ***8c***. Yield 0.99 g (68%), m.p. 296–298°C. IR (KBr) cm^−1^: 3675, 3566, 3021, 2855, 1678, 1602, 1521, 1451, 1388, 1285, 1056, 959. MS (*m/z*, %): 351 (M^+^, 0.2), 243 (8), 215 (7), 202 (100), 143 (13), 131 (5), 109 (15), 102 (5), 90 (13), 77 (5), 63 (13), 51 (21). Anal. Calcd. for C_17_H_13_N_5_O_2_S: C, 58.11; H, 3.73; N, 19.93. Found: C, 58.10; H, 3.72; N, 19.86%.

*Compound* ***8d***. Yield 0.93 g (59%), m.p. 260–262°C. IR (KBr) cm^−1^: 3677, 3075, 1695, 1588, 1503, 1464, 1341, 1274, 1066, 952. MS (*m/z*, %): 380 (M^+^, 0.5), 243 (24), 216 (80), 202 (21), 188 (32), 143 (28), 134 (18), 102 (22), 89 (100), 76 (75), 63 (88), 51 (97). Anal. Calcd. for C_17_H_12_N_6_O_3_S: C, 53.68; H, 3.18; N, 22.09. Found: C, 53.87; H, 3.17; N, 22.16%. 

#### 4.1.3. Preparation of Alkyl-2-([1,2,4]triazolo[4,3-a]quinoxalin-4-ylthio)acetate **(9a–e)**

General procedure: Equimolar quantities of the potassium salt **7** (1.2 g, 0.01 mol) and alkyl chloroacetate (0.01 mol) in DMF (20 mL) were heated on water bath for 3 h. The reaction mixture was poured onto ice water (200 mL), and stirred for 30 min. The obtained white solid was filtered and crystallized from aqueous ethanol. 

*Compound* ***9a***. Yield 0.95 g (70%), m.p. 200–202°C. IR (KBr) cm^−1^: 3454, 1722, 1464, 1316, 1200, 1060, 982, 952. (*m/z*, %): 274 (M^+^, 10), 243 (4), 215 (100), 188 (55), 170 (15), 161 (26), 143 (36), 122 (16), 116 (13), 102 (11), 76 (8), 58 (46). ^1^H NMR (DMSO-*d*_6_, 500 MHz) **δ**: 3.35 (s, 3H), 4.27 (s, 2H), 7.62 (m, 2H), 7.83 (d, 1H, *J* = 7.65 Hz), 8.32 (d, 1H, *J* = 8.6 Hz), 10.09 (s, 1H, triazolo proton). Anal. Calcd. for C_18_H_13_N_5_O_3_S: C, 52.54; H, 3.67; N, 20.43. Found: C, 52.73; H, 3.66; N, 20.49%.

*Compound* ***9b***. Yield 1.1 g (79%), m.p. 174–176°C. IR (KBr) cm^−1^: 3099, 2966, 2346, 1727, 1641, 1486, 1388, 1171, 1035, 951. (*m/z*, %): 288 (M^+^, 16), 243 (19), 215 (100), 188 (61), 170 (25), 161 (39), 143 (42), 121 (18), 115 (18), 102 (14), 75 (21), 50 (10). ^1^H NMR (CDCl_3_, 300 MHz) **δ**: 1.29 (t, 3H, *J* = 4.5 Hz), 4.18 (q, 2H, *J* = 3 Hz), 4.30 (s, 2H), 7.59 (m, 2H), 7.88 (d, 1H, *J* = 3.6 Hz), 7.94 (d, 1H, *J* = 3.6 Hz), 9.28 (s, 1H, triazolo proton). Anal. Calcd. for C_13_H_12_N_4_O_2_S: C, 54.15; H, 4.20; N, 19.43. Found: C, 54.34; H, 4.21; N, 19.37%. 

*Compound* ***9c***. Yield 0.99 g (66%), m.p. 150–152°C. IR (KBr) cm^−1^: 3423, 3069, 2966, 1743, 1641, 1390, 1299, 1167, 1063, 960. (*m/z*, %): 303 (M + 1, 40), 302 (M^+^, 16), 243 (14), 215 (100), 188 (26), 170 (13), 161 (9), 143 (16), 122 (12), 116 (8), 102 (7), 90 (13), 63 (4). Anal. Calcd. for C_14_H_14_N_4_O_2_S: C, 55.61; H, 4.67; N, 18.53. Found: C, 55.74; H, 4.65; N, 18.49%. 

*Compound* ***9d***. Yield 1.31 g (83%), m.p. 170–172°C. IR (KBr) cm^−1^: 3446, 2934, 2725, 1728, 1626, 1552, 1468, 1368, 1171, 1063, 953. ^1^H NMR (CDCl_3_, 300 MHz) **δ**: 0.77 (t, 3H, *J* = 7.2 Hz), 1.21 (sextet, 2H, *J* = 7.2 Hz), 1.51 (p, 2H, *J* = 6.3 Hz), 4.09 (t, 2H, *J* = 6.3 Hz), 4.29 (s, 2H), 7.30 (m, 2H), 8.18 (d, 1H, *J* = 6 Hz), 8.35 (d, 1H, *J* = 5.4 Hz), 9.96 (s, 1H). Anal. Calcd. for C_15_H_16_N_4_O_2_S: C, 56.94; H, 5.10; N, 17.71. Found: C, 56.92; H, 5.07; N, 17.69%.

*Compound* ***9e***. Yield 1.23 g (78%), m.p. 175–177°C. IR (KBr) cm^−1^: 3052, 2976, 2926, 1738, 1466, 1306, 1146, 1068, 956. (*m/z*, %): 316 (M^+^, 3), 260 (11), 243 (10), 216 (46), 188 (23), 143 (7), 121 (4), 102 (3), 90 (7), 56 (100). ^1^H NMR (DMSO-*d*_6_, 500 MHz) **δ**: 1.33 (s, 9H), 4.13 (s, 2H), 7.64 (m, 2H), 7.85 (d, 1H, *J* = 7.65 Hz), 8.33 (d, 1H, *J* = 7.65 Hz). Anal. Calcd. for C_15_H_16_N_4_O_2_S: C, 56.94; H, 5.10; N, 17.71. Found: C, 56.72; H, 5.09; N, 17.63%. 

#### 4.1.4. Preparation of 2-([1,2,4]Triazolo[4,3-a]quinoxalin-4-ylthio)acetic Acid Hydrazide **(10)**

Compound **9b** (3.3 g, 0.01 mol) was dissolved in absolute ethanol (50 mL) and treated with hydrazine hydrate (95%, 20 mL). The reaction mixture was stirred well and heated to 50°C for two hours, then cooled and treated with water (200 mL). The solid thus obtained was filtered, washed with water, dried, and then crystallized from glacial acetic acid to give 2.85 g (91%), m.p. > 360°C, IR (KBr) cm^−1^: 3412, 3196, 3072, 1630, 1488, 1388, 1250, 1164, 1058, 954. MS (*m/z*, %): 274 (M^+^, 4), 215 (7), 202 (22), 169 (26.86), 143 (16), 128 (47), 102 (22), 76 (100), 51 (68). ^1^H NMR (DMSO-*d*_6_, 500 MHz) **δ**: 4.08 (s, 2H), 7.62 (m, 2H), 7.92 (d, 1H, *J* = 7.6 Hz), 8.31 (d, 1H, *J* = 7.6 Hz), 10.05 (s, 1H). Anal. Calcd. for C_11_H_10_N_6_OS: C, 48.17; H, 3.67; N, 30.64. Found: C, 48.03; H, 3.68; N, 30.74%. 

#### 4.1.5. Preparation of 1-[2-([1,2,4]Triazolo[4,3-a]quinoxalin-4-ylthio)]acetyl-3,5-dimethylpyrazole **(11)**

A mixture of **10** (1 g, 0.0028 mol) and acetylacetone (0.36 g, 0.0028 mol) in absolute ethanol (20 mL) was heated at 80°C on a water bath for 7 h. The reaction mixture was cooled and poured onto water, and the formed precipitate was filtered and crystallized from ethanol to give 0.78 g (64%), m.p. 236–238°C, IR (KBr) cm^−1^: 3078, 1721, 1572, 1488, 1321, 1230, 1133, 1031, 956. MS (*m/z*, %): 338 (M^+^, 0.06), 265 (35), 249 (4), 237 (39), 222 (24), 195 (11), 169 (10), 143 (14), 115 (11), 102 (18), 76 (34), 51 (100). ^1^H NMR (DMSO-*d*_6_, 500 MHz) **δ**: 2.21 (s, 3H), 3.26 (s, 3H), 4.26 (s, 2H), 6.23 (s, 1H, pyrazole proton), 7.67 (t, 1H, *J* = 7.65 Hz), 7.77 (t, 1H, *J* = 7.65 Hz), 7.98 (d, 1H, *J* = 8.4 Hz), 8.43 (d, 1H, *J* = 7.65 Hz), 10.17 (s, 1H, triazolo proton). Anal. Calcd. for C_16_H_14_N_6_OS: C, 56.79; H, 4.17; N, 24.84. Found: C, 56.99; H, 4.15; N, 24.91%. 

#### 4.1.6. Preparation of 1-[2-([1,2,4]Triazolo[4,3-a]quinoxalin-4-ylthio)acetyl]-4,5-dihydro-5-methylpyrazol-5-one **(12)**

A mixture of **10** (1 g, 0.0028 mol) and ethyl acetoacetate (0.47 g, 0.0028 mol) in dioxane (20 mL) was heated under reflux for 5 h. The reaction mixture was cooled and poured onto water, and the formed precipitate was filtered and then crystallized from dioxane to give 0.37 g (30%), m.p. 242–244°C, IR (KBr) cm^−1^: 3412, 3192, 3069, 1635, 1491, 1388, 1248, 1180, 1062, 970. MS (*m/z*, %): 340 (M^+^, 2), 221 (3), 201 (2), 135 (6), 121 (3), 69 (48), 55 (100). ^1^H NMR (DMSO-*d*_6_, 300 MHz) **δ**: 3.43 (s, 3H), 4.38 (s, 2H, –S–CH_2_–), 5.61 (s, 2H, CH_2_ of pyrazole ring), 7.41–8.39 (m, 4H), 10.13 (s, 1H, triazolo proton). Anal. Calcd. for C_15_H_12_N_6_O_2_S: C, 52.93; H, 3.55; N, 24.69. Found: C, 53.14; H, 3.56; N, 24.72%. 

#### 4.1.7. Preparation of 5-{([1,2,4]Triazolo[4,3-a]quinoxalin-4-ylthio)methyl}-1,3,4-oxadiazole-2-thiol **(13)**

A mixture of **10** (1 g, 0.01 mol), KOH (0.01 mol), and CS_2_ (20 mL) in DMF (20 mL) was heated under reflux until the H_2_S ceased to evolve (about 5 h). The excess solvent was removed by distillation, the residue was stirred with water and filtered, and the filtrate was acidified with 10% HCl. The precipitated solid was filtered, washed with water, and crystallized from ethanol to give 0.9 g (86%), m.p. > 360°C, IR (KBr) cm^−1^: 3428, 3111, 2896, 1642, 1467, 1396, 1257, 1098, 952. MS (*m/z*, %): 316 (M^+^, 6), 242 (10), 215 (6), 210 (21), 202 (81), 143 (11), 121 (7), 105 (17), 77 (18), 52 (100). ^1^H NMR (DMSO-*d*_6_, 500 MHz) **δ**: 4.79 (s, 2H), 7.59 (m, 2H), 8.28 (d, 1H, *J* = 7.65 Hz), 9.91 (d, 1H, *J* = 7.56 Hz), 10.10 (s, 1H, triazolo proton). Anal. Calcd. for C_12_H_8_N_6_OS_2_: C, 45.56; H, 2.55; N, 26.56. Found: C, 45.72; H, 2.54; N, 26.47%. 

#### 4.1.8. Preparation of 1-([1,2,4]Triazolo[4,3-a]quinoxalin-4-ylthio)acetyl-4-cyclohexylsemicarbazide **(14)**

Compound **10** (1 g, 0.006 mol) and cyclohexyl isocyanate (0.45 g, 0.006 mol) in benzene were heated under reflux for 24 h. After cooling, the precipitate was collected and crystallized from ethanol to give 1.1 g (80%), m.p. > 360°C, IR (KBr) cm^−1^: 3390, 3091, 2929, 1669, 1534, 1497, 1334, 1239, 1175, 954. ^1^H NMR (DMSO-*d*_6_, 500 MHz) **δ**: 0.97–1.78 (m, 11H), 4.15 (s, 2H), 7.08–7.29 (m, 4H), 9.57 (s, 1H, triazolo proton). Anal. Calcd. for C_18_H_21_N_7_O_2_S: C, 54.12; H, 5.30; N, 24.54. Found: C, 54.31; H, 5.30; N, 24.61%. 

#### 4.1.9. Preparation of 1-([1,2,4]Triazolo[4,3-a]quinoxalin-4-ylthio)acetyl-4-substituted Thiosemicarbazide ** (15a, b)**

A mixture of compound **10** (1 g, 0.006 mol) and the appropriate thiocyanate (0.006 mol) in 20 mL absolute ethanol was heated under reflux for 3 h. After cooling, the precipitate was collected and crystallized from dioxane to obtain compounds (**15a-b**). 

*Compound* ***15a***. Yield 0.30 g (30%), m.p. 238–240°C, IR (KBr) cm^−1^: 3495, 3066, 1701, 1639, 1522, 1316, 1227, 1051, 955. MS (*m/z*, %): 361 (M+1, 8), 241 (9), 211 (9), 201 (8.36), 184 (31), 157 (25), 122 (9), 115 (14), 102 (15), 77 (13), 63 (100). ^1^H NMR (DMSO-*d*_6_, 300 MHz) **δ**: 0.99 (t, 3H, *J* = 6.9 Hz), 1.06 (q, 2H, *J* = 6.9 Hz), 4.29 (s, 2H), 7.67 (m, 2H), 7.96 (d, 1H, *J* = 7.2 Hz), 8.35 (d, 1H, *J* = 7.5 Hz), 10.11 (s, 1H, triazolo proton). Anal. Calcd. for C_14_H_15_N_7_OS_2_: C, 46.52; H, 4.18; N, 27.13. Found: C, 46.68; H, 4.19; N, 27.21%. 

*Compound* ***15b***. Yield 1 g (70%), m.p. 300–302°C, IR (KBr) cm^−1^: 3499, 3173, 2988, 1701, 1688, 1526, 1329, 1246, 1061, 960. ^1^H NMR (DMSO-*d*_6_, 500 MHz) **δ**: 4.21 (s, 2H), 7.62–7.31 (m, 4H), 7.33–7.65 (m, 4H), 10.02 (s, 1H, triazolo proton). Anal. Calcd. for C_18_H_15_N_7_OS_2_: C, 52.80; H, 3.69; N, 23.94. Found: C, 52.99; H, 3.70; N, 24.01%.

#### 4.1.10. Preparation of 2-([1,2,4]Triazolo[4,3-a]quinoxalin-4-ylthio)-N′-acetylacetic Acid Hydrazide **(16)**

The hydrazide **10** (1 g, 2 mmol) was warmed with acetic anhydride (5 mL) for 1 h, and then the mixture was allowed to attain room temperature. The deposited red solid was filtered, washed with petroleum ether (60–80°C), and crystallized from ethanol to afford 0.5 g (45%), m.p. 200–202°C, IR (KBr) cm^−1^: 3433, 3130, 3011, 1727, 1670, 1499, 1370, 1238, 1022, 976. MS (*m/z*, %): 316 (M^+^, 0.1), 242 (36), 200 (86), 170 (100), 143 (89), 116 (50), 75 (48), 50 (34). ^1^H NMR (DMSO-*d*_6_, 300 MHz) **δ**: 2.40 (s, 3H), 4.20 (s, 2H), 7.69 (m, 2H), 8.04 (d, 1H, *J* = 7.8 Hz), 8.43 (d, 1H, *J* = 7.8 Hz), 10.19 (s, 1H, triazolo proton). Anal. Calcd. for C_13_H_12_sN_6_O_2_S: C, 49.36; H, 3.82; N, 26.57. Found: C, 49.53; H, 3.83; N, 26.65%. 

#### 4.1.11. Preparation of N-Arylidene Derivative of 2-([1,2,4]Triazolo[4,3-a]quinoxalin-4-ylthio)acetic Acid Hydrazide **(17a–f)**

General procedure: a mixture of 1.0 g (0.028 mol) of compound **10** and the appropriate aromatic aldehyde (0.029 mol) in absolute ethanol (20 mL) and catalytic amount of glacial acetic acid (2 mL) was refluxed for 3-4 h, and the reaction mixture was then poured onto water. The solid so obtained was filtered and crystallized from acetic acid to obtain compounds **17a**–**f**. 

*Compound* ***17a***. Yield 0.17 g (46%), m.p. 350–352°C, IR (KBr) cm^−1^: 3438, 3116, 2925, 1716, 1645, 1480, 1388, 1262, 1169. ^1^H NMR (DMSO-*d*_6_, 500 MHz) **δ**: 4.65 (s, 2H), 7.57–7.63 (m, 5H), 8.07 (s, 1H), 8.09–8.36 (m, 4H), 9.92 (s, 1H, triazolo proton). Anal. Calcd. for C_18_H_14_N_6_OS: C, 59.88; H, 3.89; N, 23.19. Found: C, 59.86; H, 3.90; N, 23.26%. 

*Compound* ***17b***. Yield 0.16 g (38%), m.p. 282–284°C, IR (KBr) cm^−1^: 3450, 3097, 1708, 1626, 1478, 1389, 1240, 1045, 955. ^1^H NMR (DMSO-*d*_6_, 300 MHz) **δ**: 1.91 (s, 3H), 5.59 (s, 2H), 7.13–7.34 (m, 4H), 7.68–7.89 (m, 4H), 8.54 (s, 1H), 10.13 (s, 1H, triazolo proton). Anal. Calcd. for C_19_H_16_N_6_OS: C, 60.62; H, 4.28; N, 22.33. Found: C, 60.88; H, 4.29; N, 22.40%. 

*Compound* ***17c***. Yield 0.30 g (69%), m.p. 274–276°C, IR (KBr) cm^−1^: 3340, 3192, 3054, 1607, 1471, 1378, 1237, 1160, 1062, 962. MS (*m/z*, %): 378 (M^+^, 7), 302 (7), 243 (7), 215 (10.32), 201 (16), 143 (19), 120 (30), 102 (16), 76 (28), 51 (100). ^1^H NMR (DMSO-*d*_6_, 500 MHz) **δ**: 4.65 (s, 2H), 6.75–6.85 (m, 4H), 7.13 (t, 1H, *J* = 7.65 Hz), 7.33 (t, 1H, *J* = 7.65 Hz), 8.01 (d, 1H, *J* = 8.4 Hz), 8.32 (d, 1H, *J* = 8.4 Hz), 8.47 (s, 1H), 10.04 (s, 1H, triazolo proton). Anal. Calcd. for C_18_H_14_N_6_O_2_S: C, 57.13; H, 3.73; N, 22.21. Found: C, 57.35; H, 3.72; N, 22.27%.

*Compound* ***17d***. Yield 0.32 g (73%), m.p. 288–290°C, IR (KBr) cm^−1^: 3435, 3101, 3054, 1622, 1457, 1396, 1266, 1193, 1058, 958. ^1^H NMR (DMSO-*d*_6_, 500 MHz) **δ**: 4.29 (s, 2H), 6.83–6.91 (m, 4H), 7.22–7.32 (m, 4H), 9.00 (s, 1H), 10.03 (s, 1H, triazolo proton). Anal. Calcd. for C_18_H_14_N_6_O_2_S: C, 57.13; H, 3.73; N, 22.21. Found: C, 57.10; H, 3.69; N, 22.15%. 

*Compound* ***17e***. Yield 0.47 g (92%), m.p. 290–292°C, IR (KBr) cm^−1^: 3450, 3093, 1629, 1482, 1349, 1226, 1084, 955, MS (*m/z*, %): 396 (M^+^, 0.04), 398 (0.03), 361 (0.05), 322 (5), 285 (0.04), 243 (4), 215 (6), 201 (6.02), 185 (100), 143 (7), 123 (34), 102 (57), 76 (45), 50 (89). ^1^H NMR (DMSO-*d*_6_, 300 MHz) **δ**: 4.73 (s, 2H), 7.20–7.39 (m, 4H), 7.48–7.69 (m, 4H), 8.49 (s, 1H), 9.95 (s, 1H, triazolo proton). Anal. Calcd. for C_18_H_13_ClN_6_OS: C, 54.48; H, 3.30; N, 21.18. Found: C, 54.62; H, 3.30; N, 21.24%.

*Compound* ***17f***. Yield 0.54 g (86%), m.p. 290–292°C, IR (KBr) cm^−1^: 3420, 3328, 3106, 1620, 1476, 1371, 1244, 1158, 1025, 952. MS (*m/z*, %): 431 (M^+^, 0.03), 395 (0.04), 321 (5), 243 (0.6), 215 (1), 211 (5), 185 (100), 143 (8), 123 (14), 76 (3), 51 (6). Anal. Calcd. for C_18_H_12_C_12_N_6_OS: C, 50.13; H, 2.80; N, 19.49. Found: C, 50.31; H, 2.79; N, 19.43%. 

## 5. Conclusion

In the present investigation, 16 new 4-substituted [1,2,4]triazolo[4,3-*a*]quinoxaline derivatives were synthesized and characterized by spectral analysis. Few of those derivatives were screened for anticonvulsant activity by PTZ animal model. Compounds **14** and **15b** exhibited the highest activity which is comparable to the standard. The activity may be explained by the presence of substituents on position 4 of the condensed heterocyclic system containing quinoxaline, fused to triazole at 1,2 positions in the backbone structure of title compounds. The electronic factors exerted by the substituents and the hydrophobic nature of phenyl nucleus in the title compounds influenced the activity.

## Figures and Tables

**Table 1:** Anticonvulsant activity of the tested compounds.

Drug/test compound	Dose mg/Kg	No. of mice	No. of mice protected	Protection %	ED50% mg/kg	Mol. Wt.	ED50% mmol/kg	Relative potency to phenobarbitone sodium
Phenobarbitone sodium	6.25 12.5 25	6 6 6	1 3 6	16.66 50 100	12.5	254	0.049	1.00
** 8a**	20 40 80	6 6 6	0 3 5	0 50 66.66	40	335	0.119	0.41
**9e**	20 40 80	6 6 6	1 3 5	16.66 50 83.33	40	316	0.126	0.38
**10**	20 40 80	6 6 6	1 2 3	16.66 33.33 50	80	274	0.291	0.16
**12**	20 40 80	6 6 6	1 3 5	16.66 50 83.33	40	340	0.117	0.41
**14**	20 40 80	6 6 6	2 4 5	33.33 66.66 83.33	30	399	0.075	0.65
**15a**	20 40 80	6 6 6	2 4 5	33.33 66.66 83.33	30	361	0.083	0.58
**15b**	20 40 80	6 6 6	2 4 5	33.33 66.66 84.33	30	409	0.073	0.66
**16**	20 40 80	6 6 6	2 4 5	33.33 66.66 83.33	30	316	0.094	0.51
**17a**	20 40 80	6 6 6	0 3 5	0 50 66.66	40	362	0.110	0.44
**17d**	20 40 80	6 6 6	1 3 5	16.66 50 66.66	40	378	0.105	0.46
**17e**	20 40 80	6 6 6	0 1 3	0 16.66 50	80	396	0.202	0.24
